# Comparison of Doppler Flow Velocity and Thermodilution Derived Indexes of Coronary Physiology

**DOI:** 10.1016/j.jcin.2022.03.015

**Published:** 2022-05-23

**Authors:** Ozan M. Demir, Coen K.M. Boerhout, Guus A. de Waard, Tim P. van de Hoef, Niket Patel, Marcel A.M. Beijk, Rupert Williams, Haseeb Rahman, Henk Everaars, Oxford Acute Myocardial Infarction (OxAMI) Study, Rajesh K. Kharbanda, Paul Knaapen, Niels van Royen, Jan J. Piek, Divaka Perera

**Affiliations:** a British Heart Foundation Centre of Excellence and National Institute for Health Research Biomedical Research Centre at the School of Cardiovascular Medicine and Sciences, King’s College London, London, United Kingdom; b Department of Clinical and Experimental Cardiology, Amsterdam University Medical Center, University of Amsterdam, Amsterdam, the Netherlands; c Department of Cardiology, VU University Medical Center, Amsterdam, the Netherlands; d Oxford Heart Centre, Oxford University Hospitals, Oxford, United Kingdom; e Department of Cardiology, Radboud University Medical Center, Nijmegen, the Netherlands

**Keywords:** coronary flow reserve, hyperemic microvascular resistance, index of microvascular resistance, microvascular dysfunction, ACS, acute coronary syndrome, APV, average peak velocity, CCS, chronic coronary syndrome, CFR, coronary flow reserve, CFR_Doppler_, Doppler-derived coronary flow reserve, CFR_thermo_, thermodilution-derived coronary flow reserve, FFR, fractional flow reserve, hMR, hyperemic microvascular resistance, IMR, index of microvascular resistance, PET, positron emission tomography, ROC, receiver-operating characteristic, T_mn_, mean transit time

## Abstract

**Objectives:**

The aim of this study was to compare Doppler flow velocity and thermodilution-derived indexes and to determine the optimal thermodilution-based diagnostic thresholds for coronary flow reserve (CFR).

**Background:**

The majority of clinical data and diagnostic thresholds for flow-based indexes are derived from Doppler measurements, and correspondence with thermodilution-derived indices remain unclear.

**Methods:**

An international multicenter registry was conducted among patients who had coronary flow measurements using both Doppler and thermodilution techniques in the same vessel and during the same procedure.

**Results:**

Physiological data from 250 vessels (in 149 patients) were included in the study. A modest correlation was found between thermodilution-derived CFR (CFR_thermo_) and Doppler-derived CFR (CFR_Doppler_) (*r*^2^ = 0.36; *P* < 0.0001). CFR_thermo_ overestimated CFR_Doppler_ (mean 2.59 ± 1.46 vs 2.05 ± 0.89; *P* < 0.0001; mean bias 0.59 ± 1.24 by Bland-Altman analysis), the relationship being described by the equation CFR_thermo_ = 1.04 × CFR_Doppler_ + 0.50. The commonly used dichotomous CFR_thermo_ threshold of 2.0 had poor sensitivity at predicting a CFR_Doppler_ value <2.5. The optimal CFR_thermo_ threshold was 2.5 (sensitivity 75.54%, specificity 81.25%). There was only a weak correlation between hyperemic microvascular resistance and index of microvascular resistance (*r*^2^ = 0.19; *P* < 0.0001), due largely to variation in the measurement of flow by each modality. Forty-four percent of patients were discordantly classified as having abnormal microvascular resistance by hyperemic microvascular resistance (≥2.5 mm Hg · cm^−1^ · s) and index of microvascular resistance (≥25).

**Conclusions:**

CFR calculated by thermodilution overestimates Doppler-derived CFR, while both parameters show modest correlation. The commonly used CFR_thermo_ threshold of 2.0 has poor sensitivity for identifying vessels with diminished CFR, but using the same binary diagnostic threshold as for Doppler (<2.5) yields reasonable diagnostic accuracy. There was only a weak correlation between microvascular resistance indexes assessed by the 2 modalities.

Management guided by hemodynamic evaluation of the coronary circulation has been shown to improve clinical outcomes in patients with both chronic coronary syndrome (CCS) and acute coronary syndrome (ACS) compared with management based on angiography alone.^1^^,^^2^ At present, the majority of coronary physiological assessment is limited to pressure-based indexes, although measurement of both coronary flow and pressure is indicated in several clinical scenarios^3^, ^4^, ^5^ and is supported by international guidelines.^6^ In current practice, direct coronary flow can be invasively estimated using 1 of 2 techniques: from flow velocity using a Doppler transducer or by thermodilution on the basis of transit time of a cold bolus between 2 thermistors.^7^^,^^8^ Because of ease of use, a shorter learning curve, and wider availability, there is a trend toward increasing use of thermodilution-based evaluation of the coronary circulation. However, it should be noted the vast majority of clinical data and diagnostic thresholds for flow-based indexes are derived from Doppler measurements, and the exact correspondence with thermodilution-derived indexes is unclear. The aim of this study was to compare Doppler flow velocity and thermodilution-derived indexes of coronary physiology head to head, in individual arteries, and to determine the optimal thermodilution-based binary diagnostic thresholds for coronary flow reserve (CFR).

## Methods

### Study population

This international multicenter registry included patients with paired Doppler and thermodilution flow measurements from 4 centers in Europe. All patients had coronary flow measurements with both Doppler and thermodilution techniques in the same vessel and during the same procedure.^9^, ^10^, ^11^, ^12^ Patients with CCS and those with ACS were eligible. CCS includes patients with angina in the context of obstructive or nonobstructive coronary disease. ACS was defined as a cardiac biomarker elevation in association with characteristic electrocardiographic changes and/or typical symptoms. Patient-level prospective data were collected at local institutions using an anonymized dedicated database and sent to study coordinators for analysis. The study was conducted in accordance with the Declaration of Helsinki, and ethical approval was gained at each center. All patients provided written informed consent.

### Invasive measurements

After diagnostic coronary angiography, a 5- or 6-F guiding catheter was advanced to the coronary ostium and an intracoronary bolus of 200 to 300 μg nitrates administered. Both pressure/Doppler (ComboWire XT, Phillips Volcano) and pressure/thermodilution (PressureWire X, Abbott) coronary guidewires were calibrated to fluid-filled aortic pressure, with the pressure sensor positioned between the tip of the guiding catheter and the coronary ostium, then advanced to the distal third of the vessel (>5 cm from the coronary ostia) and/or distal to stented segment in vessels that had percutaneous coronary intervention prior to physiological assessment. In the case of the ComboWire, the tip was manipulated until an optimal and stable high-quality Doppler flow signal was obtained, and Doppler flow velocity, electrocardiographic signals, aortic pressure, and distal coronary pressure were simultaneously recorded using the ComboMap system (Philips Volcano), at rest and during hyperemia. The PressureWire X was connected to a RadiAnalyzer interface (Abbott) and thermodilution performed as follows: 3 to 5 mL saline at room temperature was rapidly injected through the guiding catheter, and this process was repeated twice, yielding 3 baseline thermodilution curves. Repeat injections were performed for outlying values. Measurements were repeated after hyperemia was induced by intravenous infusion of 140 μg/kg/min adenosine or intracoronary bolus injection of 15 to 20 mg papaverine.

### Data analysis

**Figure 1:**
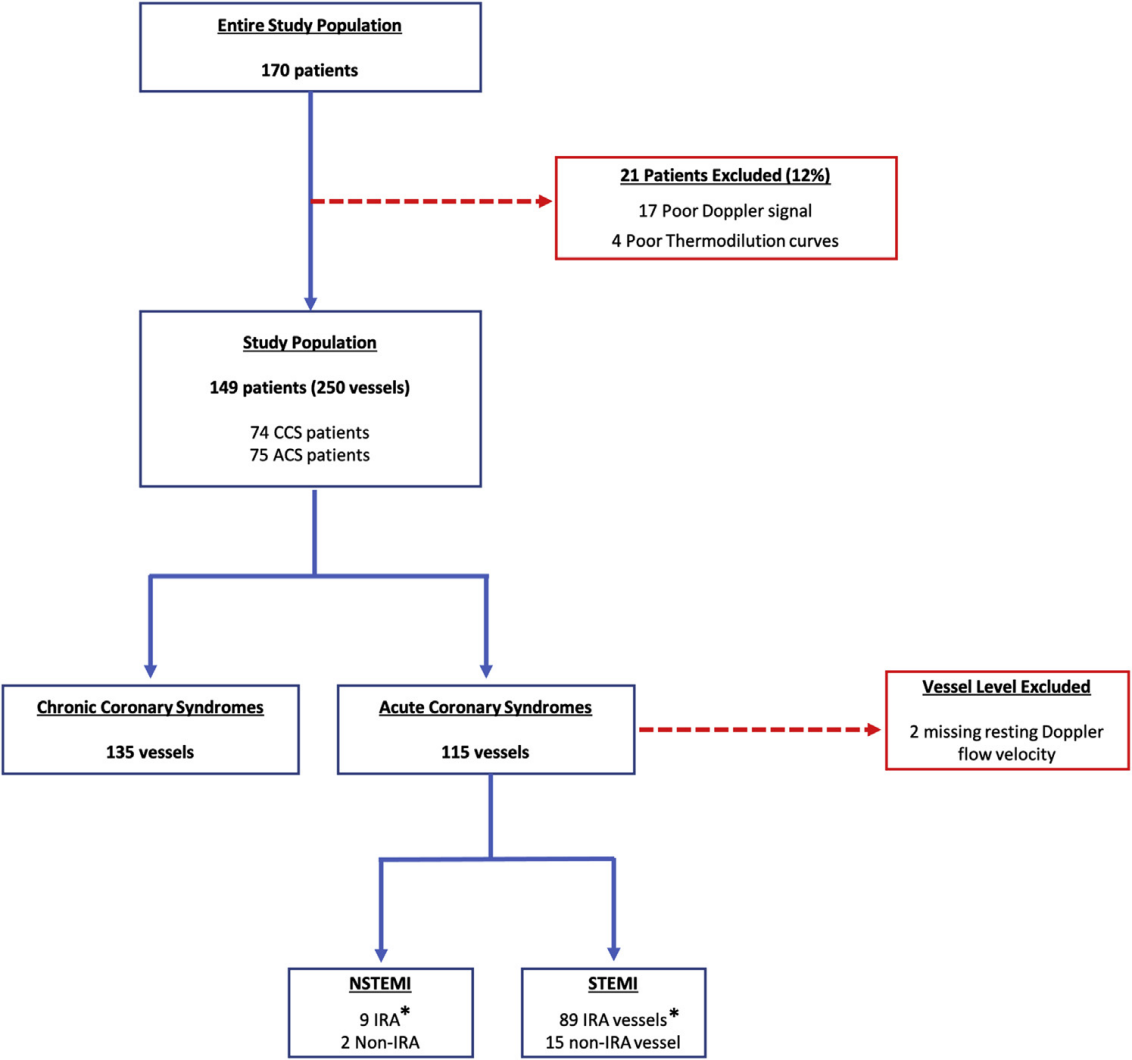
Consolidated Standards of Reporting Trials Diagram ∗Post–percutaneous coronary intervention measurements. ACS = acute coronary syndrome; CCS = chronic coronary syndrome; IRA = infarct-related artery; NSTEMI = non–ST-segment elevation myocardial infarction; STEMI = ST-segment elevation myocardial infarction.

Coronary hemodynamic data were extracted from the ComboMap and RadiAnalyzer systems for off-line analysis. Data quality was adjudicated at each center by systematic review of Doppler flow velocity traces and thermodilution curves; poor quality data were excluded from the analysis (Figure 1). Fractional flow reserve (FFR) was defined as the ratio of distal coronary to aortic pressure during maximal hyperemia.^13^ Doppler peak flow velocities were averaged over ≥3 consecutive heartbeats, to derive average peak velocity (APV). Doppler-derived CFR (CFR_Doppler_) was defined as the ratio of hyperemic to resting APV.^7^ Hyperemic microvascular resistance (hMR) was defined as the ratio between hyperemic mean distal pressure and hyperemic APV.^14^ Transit times were calculated from the thermodilution curves and mean transit time (T_mn_) was computed at rest and during hyperemia by averaging 3 transit times.^8^ Subsequently, thermodilution-derived CFR (CFR_thermo_) was calculated by dividing resting T_mn_ by hyperemic T_mn_.^15^ Index of microvascular resistance (IMR) was defined as the hyperemic mean distal pressure multiplied by hyperemic T_mn_.^15^

### Statistical analysis

Continuous variables are presented as mean ± SD or as median (IQR). Categorical variables are presented as frequency (percentage). Comparisons were analyzed using the 2-tailed paired Student’s *t*-test (for parametric measurements) and the Mann-Whitney *U* test (nonparametric measurements). The correlation between indexes was analyzed by calculating the Pearson correlation coefficient (*r*), and agreement between indexes was assessed using Bland-Altman plots of the relative differences. Variance was assessed using the Levene test (parametric) or Brown-Forsythe test (nonparametric). Receiver-operating characteristic (ROC) analysis of indexes was performed using CFR_Doppler_ <2.50 as a reference standard. Diagnostic accuracy was defined as the proportion of correctly classified patients (true positive and true negative) among all subjects (true positive, true negative, false positive, and false negative), with CFR_Doppler_ <2.50 as the reference standard.^16^ A *P* value of 0.05 was considered to indicate statistical significance. All analyses were performed using SPSS version 27.0 (IBM) and Prism version 9.1.2 (GraphPad Software).

## Results

**Figure 2:**
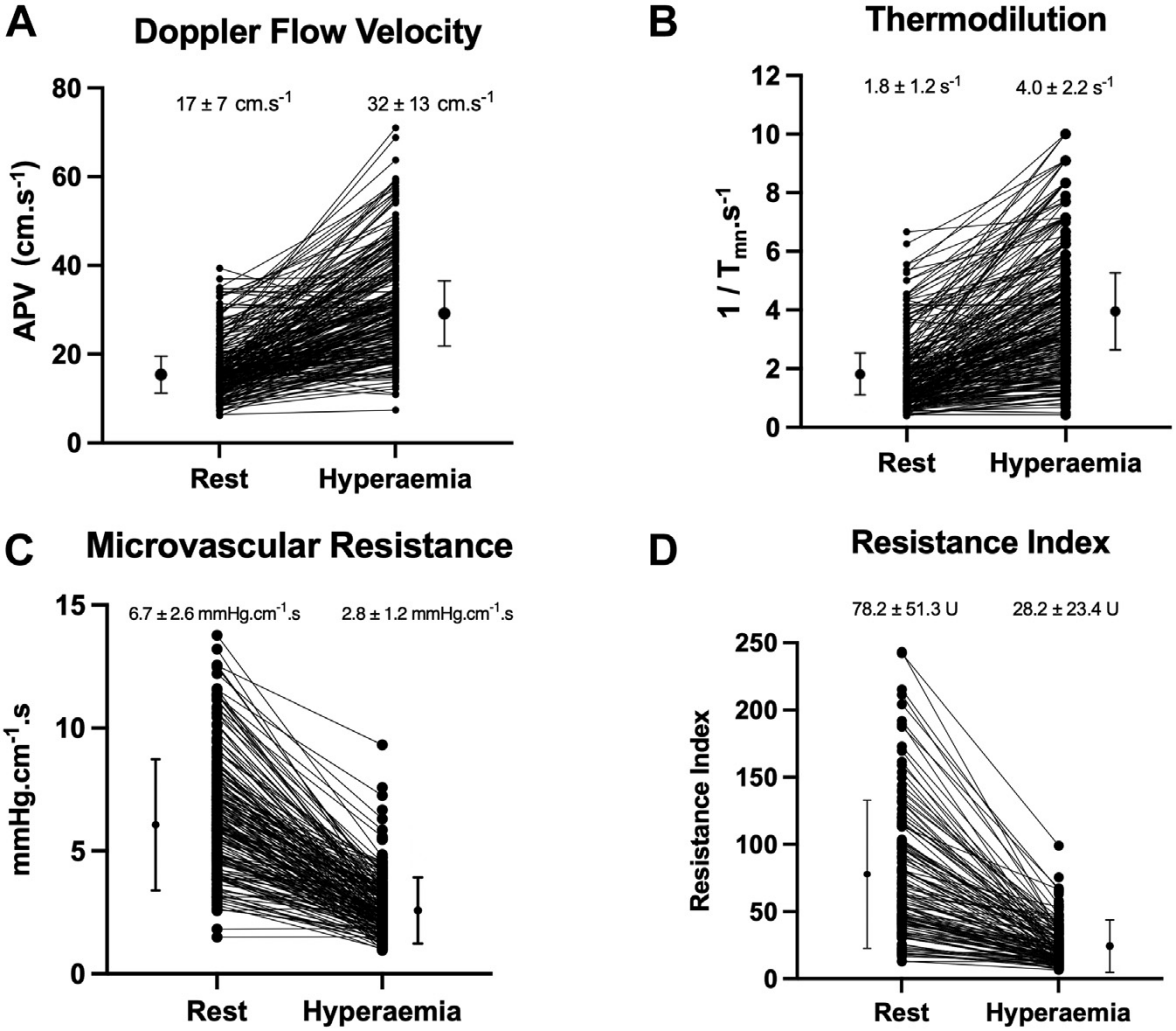
Hyperemic Responses of Individual Vessels **(A)** Doppler flow velocity. **(B)** Thermodilution. **(C)** Microvascular resistance measured by Doppler flow velocity. **(D)** Resistance index measured by thermodilution. **Numbers and middle bars** depict mean values, and **error bars** depict 95% confidence intervals. APV = average peak velocity; T_mn_ = mean transit time.

A total of 170 patients were included in the study (Figure 1). Of these, 21 patients (12%) were excluded because of poor quality data acquisition, 17 (10%) in the Doppler group and 4 (2%) in the thermodilution group. The remaining 149 patients (88%) formed the study population (mean age 60.7 ± 9.7 years, 81% men, 22% with diabetes mellitus, 50% with hypertension, 54% with hypercholesterolemia) (Table 1). Mean left ventricular ejection fraction was 47.7% ± 10.9%. In these 149 patients, 250 vessels underwent physiological evaluation. The target vessel was the left anterior descending coronary artery in 48%, the left circumflex coronary artery in 22%, and the right coronary artery in 30%. Mean FFR was 0.89 ± 0.11, and 36 vessels (14%) had hemodynamically significant stenosis (FFR ≤0.80; note that physiology was performed post-PCI in patients with coronary disease). Patients with CCS had lower FFR values compared with those with ACS (0.87 ± 0.14 vs 0.92 ± 0.06, respectively; *P* < 0.001). Figure 2 displays individual hyperemic responses as measured with Doppler flow velocity, thermodilution flow, Doppler-derived microvascular resistance, and thermodilution-derived IMR.

### Agreement between CFR_thermo_ and CFR_Doppler_

**Figure 3:**
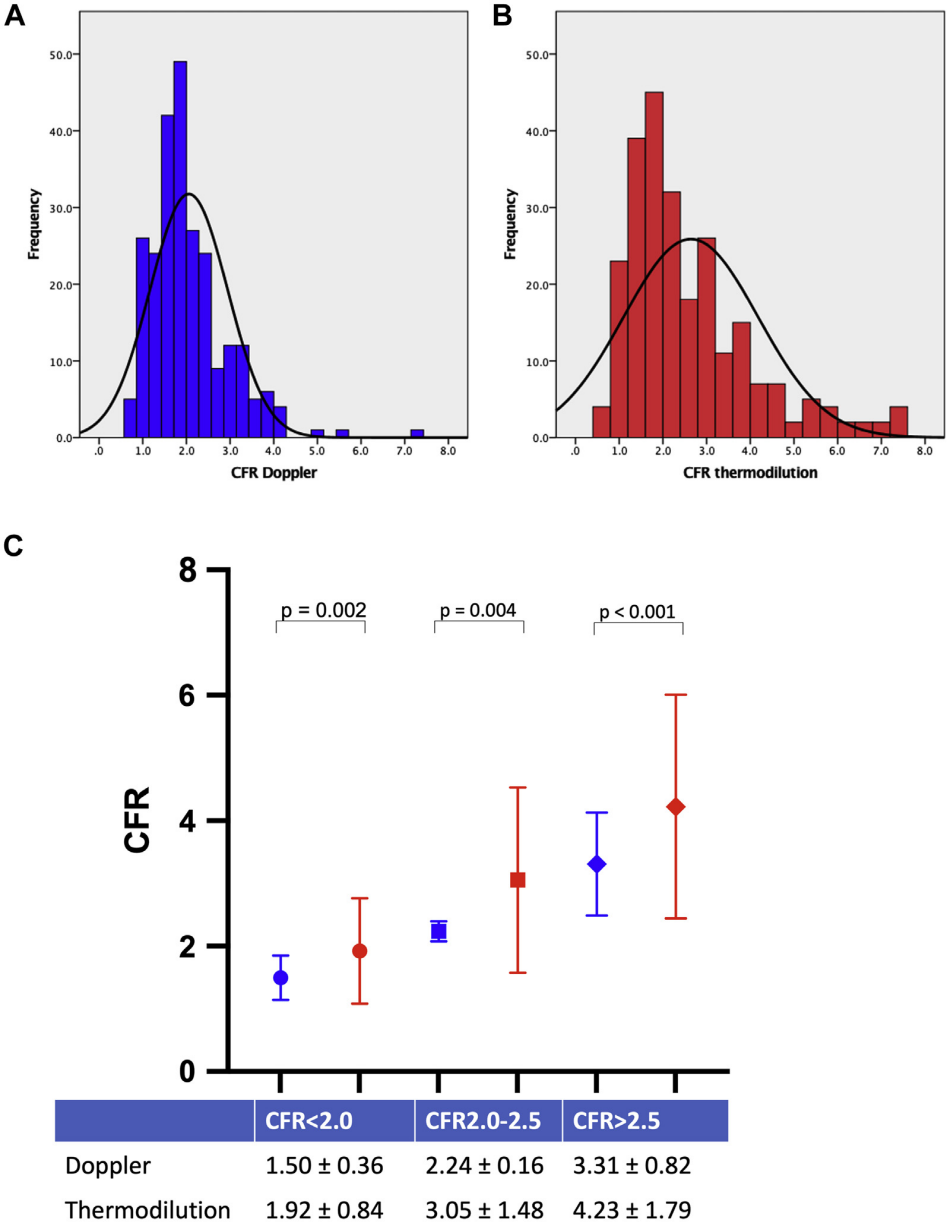
Distribution of CFR **(A)** Histogram of Doppler-derived coronary flow reserve (CFR) measurements. **(B)** Histogram of thermodilution-derived CFR measurements. **(C)** Average CFR values for prespecified range of <2.0, 2.0 to 2.5, and >2.5. ∗Statistically significant difference between groups (*P* < 0.05). **Numbers and middle bars** depict mean values, and **error bars** depict 95% confidence intervals.

**Figure 4:**
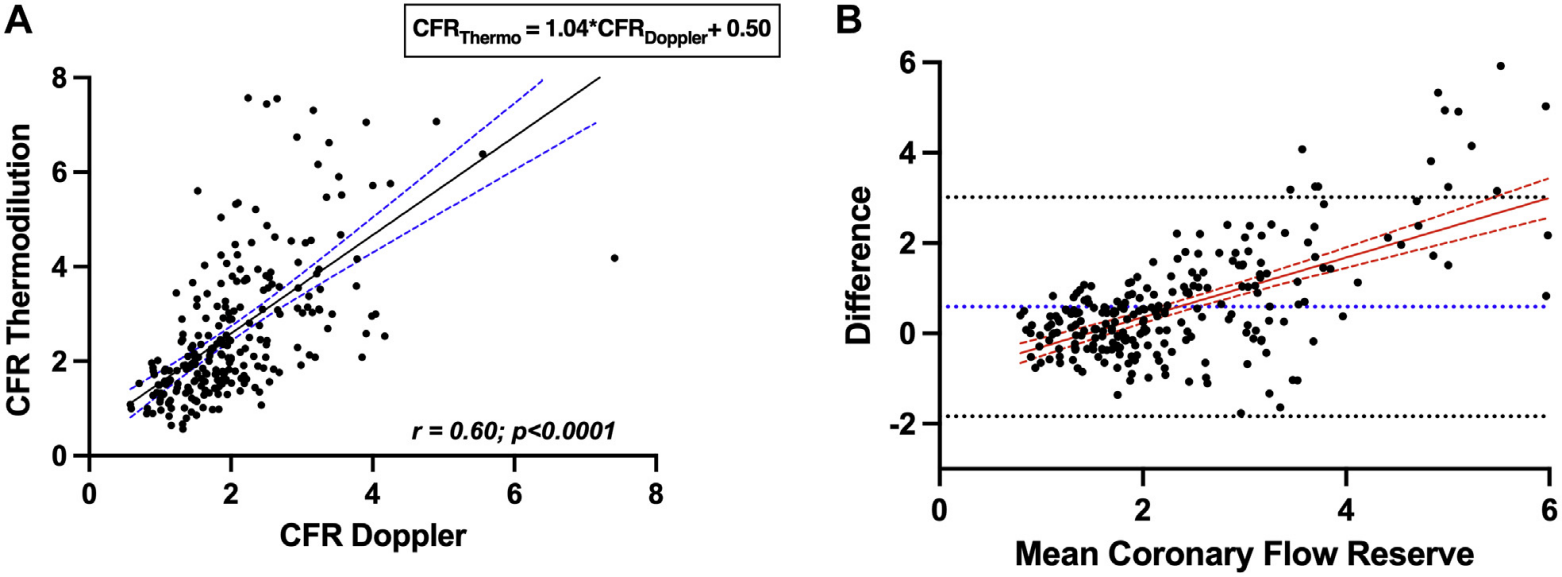
Scatterplot and Bland-Altman Plot of CFR_Doppler_ and CFR_thermo_ **(A)** Scatterplot of coronary flow reserve (CFR) measurements. The **black line** represents the line of best fit between Doppler-derived CFR (CFR_Doppler_) and thermodilution-derived CFR (CFR_thermo_). The **dashed blue lines** represent the 95% CI for the line of best fit (*r* = 0.60; *r*^2^ = 0.36; 95% CI: 0.51 to 0.67; *P* < 0.001). **(B)** Bland-Altman plot of differences against the means for CFR (CFR_thermo_ − CFR_Doppler_). The mean bias (0.59) is represented by the **dotted blue line**, and the 95% CI (−1.84 to 3.01) is represented by the **dotted black lines**. The mean difference is represented by the **red line** and the 95% CI by the **dashed red lines**.

There was wider distribution of CFR_thermo_ measurements compared with CFR_Doppler_ measurements when assessing normality of distribution (Figures 3A and 3B). Mean CFR_thermo_ was significantly higher than CFR_Doppler_ (2.59 ± 1.46 vs 2.05 ± 0.89; *P* < 0.0001). When measurements were trichotomized by CFR values (<2.0, 2.0-2.5, and >2.5) there was a significant difference between CFR_Doppler_ and CFR_thermo_ across all groups (Figure 3C); the greatest absolute difference was seen in the group with CFR >2.5 (4.23 ± 1.79 for CFR_thermo_ vs 3.31 ± 0.82 for CFR_Doppler_; *P* < 0.0001). A moderate correlation was found between CFR_thermo_ and CFR_Doppler_ (*r* = 0.60; *P* < 0.0001) (Central Illustration), and CFR_thermo_ = 1.04 × CFR_Doppler_ + 0.50 (Figure 4A). The corresponding Bland-Altman plot demonstrated a bias toward overestimation of CFR by CFR_thermo_ (0.59 ± 1.24) (Figure 4B). There was significant heteroscedasticity between CFR_thermo_ and CFR_Doppler_ measurements (Levene statistic [*F*] = 2.99). Furthermore, the mean bias was not constant throughout the range of values, and thermodilution provided higher CFR values than Doppler flow velocity in the highest range of values, and vice versa at lower values. CFR by either modality was lower in patients with ACS than in those with CCS. Subgroup analysis according to clinical status (CCS or ACS) demonstrated that the average CFR_thermo_ was significantly higher than CFR_Doppler_ in both the CCS (3.12 ± 1.61 vs 2.37 ± 0.90; *P* < 0.0001) and ACS (2.07 ± 1.26 vs 1.67 ± 0.71; *P* < 0.0001) groups. In addition, overestimation of coronary flow when using CFR_thermo_ was present irrespective of vessel interrogated.

**Central Illustration:**
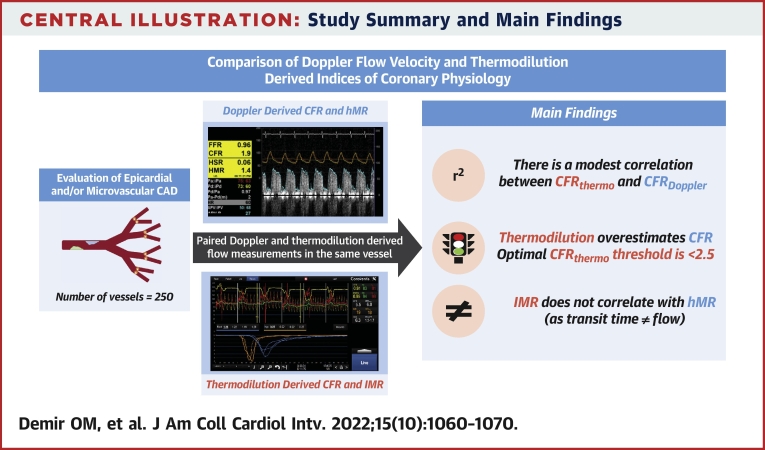
Study Summary and Main Findings CFR = coronary flow reserve; CFR_Doppler_ = Doppler-derived coronary flow reserve; CFR_thermo_ = thermodilution-derived coronary flow reserve; hMR = hyperemic microvascular resistance; IMR = index of microvascular resistance.

**Figure 5:**
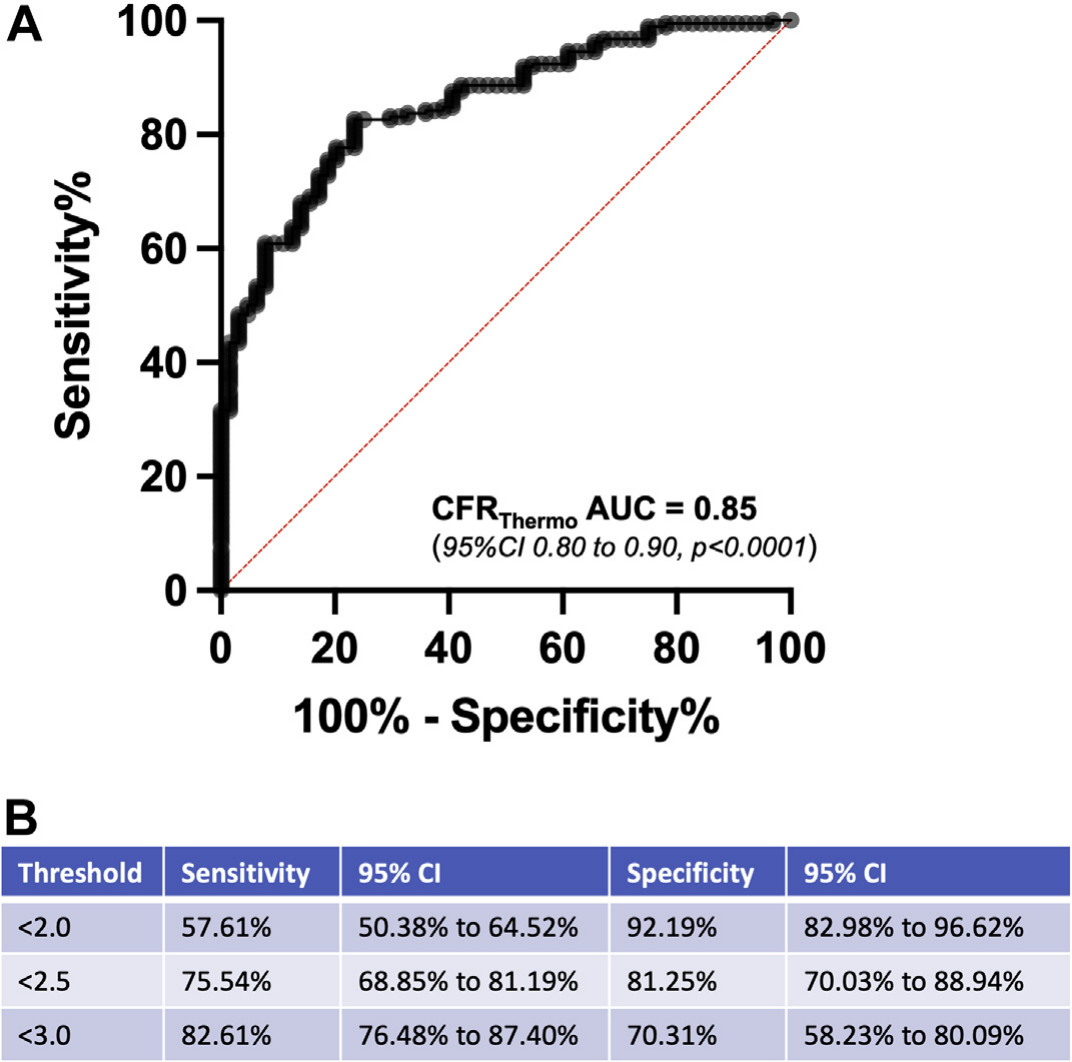
Receiver-Operating Characteristic Curves for Thermodilution-Derived CFR **(A)** Receiver-operating characteristic curves for thermodilution-derived coronary flow reserve (CFR_thermo_) in predicting Doppler-derived coronary flow reserve at a value <2.5. **(B)** Sensitivity and specificity of CFR_thermo_ at various thresholds. AUC = area under the curve.

A CFR_Doppler_ value of <2.5 was used as reference standard for ROC analysis of CFR measurements to identify clinically relevant composite dysfunction of the epicardial and microcirculation (Figure 5). The area under the ROC curve for CFR_thermo_ was 0.85 (95% CI: 0.80-0.90; *P* < 0.0001). The commonly used CFR_thermo_ threshold of <2.0 had sensitivity of 57.61% and specificity 92.19% at predicting a CFR_Doppler_ value <2.5. For CFR_thermo_ thresholds of <2.5 and <3.0, sensitivities and specificities were 75.54% and 81.25% and 82.61% and 70.31%, respectively. The diagnostic accuracy of CFR_thermo_ at thresholds of <2.0, <2.5, and <3.0 was 66% (95% CI: 62%-71%), 79% (95% CI: 73%-87%), and 81% (95% CI: 75%-88%), respectively.

### Correlation between microvascular resistance indexes

**Figure 6:**
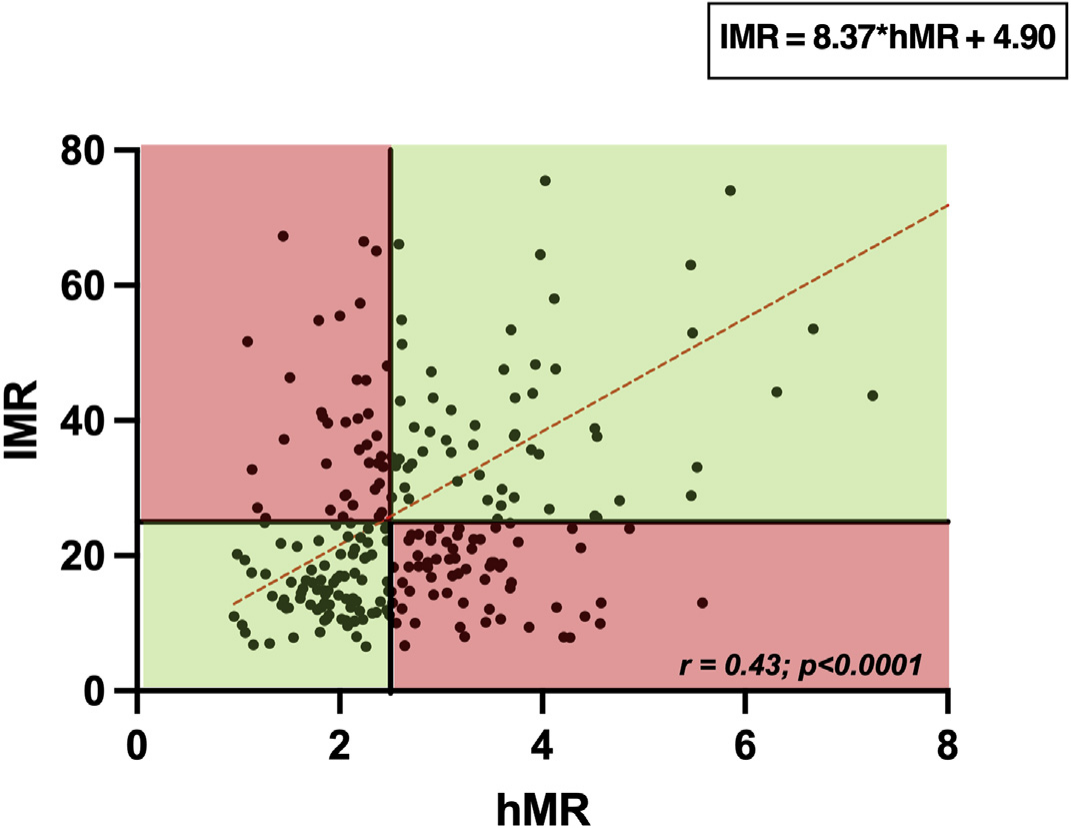
Scatterplot of hMR and IMR The **dashed red lines** represent the line of best fit (*r* = 0.43; *r*^2^ = 0.19; 95% CI: 0.32-0.52; *P* < 0.001). Quadrants shaded in **green** have concordant and **red** discordant measurements, using thresholds of hyperemic microvascular resistance (hMR) ≥2.5 and index of microvascular resistance (IMR) ≥25.

Median hMR was 2.51 mm Hg · cm^−1^ · s (IQR: 2.00-3.40 mm Hg · cm^−1^ · s), and median IMR was 22.04 U (IQR: 14.25-35.18 U). A modest correlation was found between hMR and IMR (*r* = 0.43; *P* < 0.0001) (Figure 6). When dichotomously classified by the commonly used thresholds (hMR ≥2.5 and IMR ≥25), 132 (53%) hMR and 106 (42%) IMR measurements were abnormal; discordant results were observed in 110 vessels (44%) and concordant results in 140 vessels (56%) (concordant 64 abnormal and 76 normal) (Figure 6). When subgroup analysis was performed, excluding patients with FFR <0.80 (to assess agreement of microvascular resistance indexes in patients without epicardial stenoses), only a weak correlation between hMR and IMR remained (*r* = 0.37; *P* < 0.0001) (Supplemental Figure 1). An exploratory post hoc analysis, dichotomously classifying the group by a lower hMR threshold (>1.9), is detailed in Supplemental Figure 2, with no difference to the overall conclusions.

When considering the components of microvascular resistance indexes, pressure and flow, the following correlations were found between the 2 modalities: for hyperemic Pd, *r* = 0.88, *P* < 0.0001, and hyperemic Pd_thermo_ = 0.84 × hyperemic Pd_Doppler_ + 12.20; and for hyperemic flow (APV vs T_mn_), *r* = 0.29, *P* < 0.0001, and hyperemic T_mn_ = −0.007 × hyperemic APV + 0.58.

## Discussion

As far as we are aware, ours is the largest head-to-head paired comparison of Doppler flow velocity and thermodilution-based physiological indexes in individual patients to date. The main findings of the study are that there was a modest correlation between CFR measured by Doppler and thermodilution (*r* = 0.60; *P* < 0.0001) and that overestimation by thermodilution was not uniform across the range; the degree of bias increased with CFR values, the error being most marked in patients with CFR >2.5. The sensitivity of CFR_thermo_ for detecting microvascular dysfunction was low when applying the commonly used threshold of 2.0 but improved if the same numeric threshold was used as for CFR_Doppler_ (<2.5). In contrast, minimal microvascular resistance measured by the 2 modalities correlated poorly, largely because of differences in the measures of hyperemic flow, and as a consequence, 44% patients were discordantly classified as having abnormal microvascular resistance by hMR (≥2.5 mmHg · cm^−1^ · s) and IMR (≥25).

### Correlation between CFR_Doppler_ and CFR_thermo_ measurements

Since the original clinical study by Pijls et al^8^ in 2002, which demonstrated a good correlation between Doppler and thermodilution measurements in 119 vessels (*r* = 0.80; *P* < 0.001), there have been few head-to-head comparative studies of Doppler- and thermodilution-based measurements. In that study, the relationship between the 2 indexes was expressed as CFR_thermo_ = 0.84 × CFR_Doppler_ + 0.17,^8^ which translates to a small but progressive underestimation by thermodilution of CFR_Doppler_ with increasing CFR values. For instance, according to this relationship, a CFR_Doppler_ value of 2.5 would be expected to relate to a CFR_thermo_ value of 2.27. Perhaps as a consequence, a CFR_thermo_ threshold of 2.0 has been used by many to diagnose microvascular dysfunction in clinical practice and in studies of this condition.^4^^,^^17^^,^^18^ Our results contrast with the 2002 study findings in 2 key respects. First, we found a more modest correlation between CFR_Doppler_ and CFR_thermo_ (*r* = 0.60; *P* < 0.0001). One reason for this difference may be related to the degree of epicardial coronary disease in the 2 populations; ours was a group with minimal or no coronary disease, whereas the studies by Pijls et al^8^ and Barbato et al^19^ included cohorts with much more significant coronary disease (FFR 0.53 ± 0.16 [in target vessel n = 45] and 0.75 ± 0.2, respectively). Hence, the CFR values encountered in the previous studies were much lower than in our study, which, given our finding of increased bias with progressively higher CFR values, would explain the stronger correlation reported in those studies. However, as the primary contemporary indication for measurement of CFR is in evaluating patients with angina despite minimal epicardial disease or unobstructed coronary arteries, the greatest need for diagnostic precision is around the CFR Doppler threshold of 2.5, which is better reflected by the population enrolled in the present registry.

Second, and with important clinical implications, we have shown that CFR_thermo_ overestimates CFR_Doppler_ (as opposed to underestimating the latter, as previously thought).^20^^,^^21^ We found the relationship between the 2 indexes to be CFR_thermo_ = 1.04 × CFR_Doppler_ + 0.50; accordingly, a CFR_Doppler_ value of 2.5 would correspond to a CFR_thermo_ value of 3.10. Indeed, a CFR_thermo_ threshold of 3.0 provides excellent diagnostic accuracy for detecting microvascular dysfunction (CFR_Doppler_ <2.5), although this was at the cost of specificity. In contrast, a threshold of 2.5 had similar sensitivity but much better specificity and is the diagnostic threshold we would recommend, which has the added advantage (for guidelines committees as well as catheterization laboratory teams) of being identical to the diagnostic threshold for CFR_Doppler_. This also means that the commonly used thermodilution threshold of 2.0 has suboptimal accuracy because of poor sensitivity (although this threshold is very specific). Current European Society of Cardiology guidance suggests that CFR <2.0 is diagnostic of abnormal microcirculatory function,^6^ with no distinction made between the modality of measuring CFR measurements; on the basis of our results, we believe that the recommended threshold for diagnosing microvascular dysfunction in patients with unobstructed epicardial arteries (where FFR >0.80 or non-hyperemic pressure ratio >0.89) be increased to 2.5, regardless of which modality is used. However, when used as a continuous variable, CFR_thermo_ values may need to be adjusted down, as per the aforementioned relationship, to allow meaningful comparison with CFR_Doppler_ datasets. The underlying basis driving overestimation of thermodilution-derived CFR has not been elucidated. The potential explanation of this discrepancy is the intrinsic differences in coronary blood flow measurements between Doppler and thermodilution techniques. Findings from this study suggest that the differences in hyperemic blood flow measurements are the principal driver of this discrepancy.

Although Doppler-based measurement of flow is theoretically more robust and is widely regarded as the reference standard against which other invasive and noninvasive measures of flow are evaluated, it must be acknowledged that ours is a comparison between 2 measurements without a truly independent gold standard. However, others have assessed the relationship of each modality compared with myocardial perfusion. Everaars et al^12^ assessed the correlation between CFR and myocardial perfusion using [^15^O]H_2_O positron emission tomography (PET) and reported a good correlation between CFR_Doppler_ and PET-derived CFR (*r* = 0.82; *P* < 0.001) and that, CFR_thermo_ and PET-derived CFR correlated only modestly (*r* = 0.55; *P* < 0.001). In keeping with our findings, Bland-Altman analysis in this PET study showed that CFR_thermo_ overestimated flow reserve at higher values compared with PET- and Doppler-derived measurements. It should also be noted that the optimal diagnostic threshold for Doppler-based CFR measurements may also vary with the population studied; in a population with angina but unobstructed epicardial arteries, a CFR_Doppler_ threshold of 2.5 has been shown to be optimal at detecting circumferential myocardial stress hypoperfusion by 3-T perfusion cardiac magnetic resonance imaging.^22^

### Agreement between indexes of microvascular resistance

There was only a weak correlation between hMR and IMR, driven largely by discrepancies between flow velocity and cold bolus transit time, with frequently discordant classification of patients, when applying accepted binary thresholds. These findings indicate the potential limitations of thermodilution-based indexes using bolus injections for evaluating coronary hemodynamic status, innate flow being influenced by bolus injections, with theoretically greatest impact at slower flow rates (eg, resting flow). This correlation did not improve when patients without physiologically significant epicardial disease (FFR >0.80) were excluded (Supplemental Figure 1). As far as we are aware, there have been no other direct head-to-head comparisons of hMR and IMR published to date, apart from the individual studies making up the present registry.^9^^,^^12^ Although hMR is a theoretically more robust index of microvascular resistance than IMR (as Doppler is widely regarded to be a more accurate reflection of absolute flow than transit time) and correlates better with various noninvasive and invasive measures of perfusion, the lack of a clinically applicable reference measure of microvascular resistance limits the ability to truly compare the diagnostic accuracy of these 2 indexes. The advent of absolute flow measurements, also based on the principle of thermodilution but using continuous infusion of cold saline, which in turn can be used to calculate “absolute” microvascular resistance, may allow a more robust measure of microvascular resistance than IMR, but robust validation is needed, with researchers once again hampered by the lack of a clinical gold standard for microvascular resistance.^23^

The reliance of both measures of microvascular resistance on respective estimates of flow, coupled with inherent inaccuracies of the latter, may also explain why the correlation between hMR and IMR is poorer than the correlation between CFR_Doppler_ and CFR_thermo_. Given that the theoretical assumptions that underlie derivation of flow by either modality are unlikely to be changed by the effects of adenosine on the coronary circulation, they are expected to cancel out when assessing the ratio of flow at rest and hyperemia, as applies to CFR.

How should the results of this study influence the application of hMR or IMR in clinical practice and research? First, caution should be exercised when comparing or combining cohorts characterized by either index, as they correlate poorly with each other. Second, the inaccuracies of both measures of microvascular resistance make them unsuitable to be regarded as first line or stand-alone diagnostic tests used to classify patients or stratify therapy. Furthermore, given the poor correlation between either hMR or IMR and CFR (measured by the respective modality), the former should not be regarded as physiologically equivalent to the latter, as currently enshrined in several practice guidelines.^6^^,^^20^ However, regardless of the lack of correlation between these Doppler- and thermodilution-based measures of microvascular resistance, or indeed whether they accurately reflect true microvascular resistance, the utility of each index as a biomarker for prognostication (such as IMR following myocardial infarction), disease classification (such as distinguishing structural from functional microvascular dysfunction, as diagnosed by a diminished CFR) and stratification of therapy warrants further evaluation in the future.

### Considerations for thermodilution measurements

Thermodilution measurements can be rapidly performed and are not associated with any extra costs compared with the present physiological measurement of FFR alone, and there is no need for extra hardware. In our study, a greater number of patients were excluded because of poor Doppler signal than thermodilution curve quality. Of note, exclusion frequency may be greater in general practice, as these data are from centers that are among those with the greatest volume of expertise in these measurements globally. From previous studies, Pijls et al^8^ reported suboptimal CFR_Doppler_ in 9% and CFR_thermo_ in 11% of vessels, and Barbato et al^19^ reported suboptimal CFR_Doppler_ in 31% of patients, and among those with satisfactory CFR_Doppler_, 3% had suboptimal CFR_thermo_ measurements. Adhering to a few practical steps when performing thermodilution measurements should maximize accuracy. First, guide catheter positioning needs careful consideration; it must be sufficiently engaged in the coronary artery to guarantee adequate delivery of the indicator into the vessel, and easy backflow into the aortic root is necessary to avoid mechanical influence of the injection on baseline blood flow, resulting in underestimation of CFR. Second, the guide catheter should be coaxial within the vessel to ensure that the saline boluses are sufficiently injected into the target vessel, preventing suboptimal and erroneous thermodilution curves with resultant incorrect physiological measurements. Third, the coronary guidewire sensor tip should be advanced ≥15 mm from the most distal lesion to minimize any potential interference with epicardial stenosis–induced flow disturbance, or approximately 5 cm from the ostium in patients with no epicardial coronary artery disease. Fourth, injections of room-temperature saline should be limited to 3 to 4 mL per injection using a 5-mL syringe to ensure optimal and uniform bolus.

### Study limitations

First, the centers participating in this study did not use a uniform study protocol. However, acquisition methodology for the invasive data was similar, and all centers used contemporary Doppler flow and pressure wires. Second, invasive measurements had to be excluded in 12% of patients because of poor data quality. Third, no core laboratory analysis of the invasive measurements was performed, and data used were as reported by each center. Fourth, our results may have been affected by the reproducibility of Doppler and thermodilution measurements. However, this is a recognized issue with biological measurements. In fact, previous data have demonstrated that the observed variation between repeated recordings of coronary blood flow using Doppler measurements at rest was 10.5% (95% CI: 7.7%-16.2%)^24^ and using thermodilution was 11.5% ± 7% at rest and 14.6% ± 9% during hyperemia, with 18.8% ± 11% variability of thermodilution-derived CFR.^12^ Fifth, although this study is the largest study to incorporate detailed physiological characterization by combined Doppler flow velocity and thermodilution measurement, sample size remains limited. Sixth, intravenous adenosine is accompanied by a decrease of blood pressure of approximately 10% to 15%, and therefore CFR may be underestimated by 10% to 15% if not corrected for these pressure changes.^8^ In this study, such a correction was not necessary, because CFR_thermo_ and CFR_Doppler_ were both measured with intravenous adenosine or intracoronary papaverine simultaneously and therefore were affected in the same way by hemodynamic status. Seventh, the poor-quality data acquisition with Doppler observed in approximately 10% of patients may not be extrapolated to all flow wires. Eighth, not all measurements were truly simultaneous; some were done sequentially immediately after the first measurement. Although there was a low likelihood of a change in hemodynamic conditions between recordings, as the order of measurements was not randomized, we cannot exclude systematic bias. Finally, resting conditions were awaited before repeat measurements were performed. However, we did not prespecify a mandatory time interval between measurements, which may have resulted in interaction between initial and subsequent physiological measurements. However, these measurements were performed at recognized leading centers in coronary physiology by experts in the field of coronary physiology.

## Conclusions

CFR_thermo_ correlates with, but overestimates, CFR_Doppler_. The commonly used CFR_thermo_ threshold of 2.0 has poor sensitivity for identifying vessels with diminished CFR, but using the same binary diagnostic threshold as for Doppler (<2.5) yields reasonable diagnostic accuracy. Microvascular resistance indices assessed by the 2 modalities correlate less well but may still have utility when combined with CFR or as prognostic biomarkers.

Perspectives **WHAT IS KNOWN?** Coronary bolus transit time, on the basis of thermodilution theory, was proposed as an indirect measure of coronary flow and is often used as an alternative to the reference standard Doppler-based techniques, as the latter are considered harder to use. However, the exact correspondence between these methods is unclear.

**WHAT IS NEW?** CFR_thermo_ is a modest approximation of CFR_Doppler_ and tends to overestimate the latter. As a result, the most widely used clinical threshold for CFR_thermo_ (<2.0) has poor sensitivity for identifying coronary microvascular dysfunction. Furthermore, the thermodilution-derived IMR does not correlate with Doppler-derived microvascular resistance.

**WHAT IS NEXT?** A unitary CFR threshold of 2.5, regardless of modality used, will offer better diagnostic accuracy than current practice. Research into readily applicable yet robust measures of coronary flow and microvascular resistance is both needed and warranted.

**Table 1:** Patient Characteristics and Angiographic and Hemodynamic Data

Clinical characteristics (n = 149)	
Age, y	60.7 ± 9.7
Male	120 (81)
Body mass index, kg/m^2^	27.3 ± 3.7
Diabetes mellitus	32 (22)
Hypertension	75 (50)
Hypercholesterolemia	81 (54)
Smoking history	83 (56)
Left ventricular ejection fraction, %	47.7 ± 10.9
Vessel characteristics (n = 250)	
Vessels per patient	1.68 ± 0.63
Number of vessel(s) per patient	
1	61 (41)
2	75 (50)
3	13 (9)
Target vessel	
Left anterior descending coronary artery	119 (48)
Left circumflex coronary artery	55 (22)
Right coronary artery	76 (30)
Pd/Pa	0.94 ± 0.09
Fractional flow reserve	0.89 ± 0.11

Values are mean ± SD or n (%).

## Funding Support and Author Disclosures

This work was supported by the British Heart Foundation (PG/19/9/34228), the National Institute for Health Research via the Biomedical Research Centre award to Guy’s and St Thomas’ Hospital and King’s College London, and the National Institute for Health Research Oxford Biomedical Research Centre. The authors have reported that they have no relationships relevant to the contents of this paper to disclose.
