# Supplementary material for: Comparison of Doppler Flow Velocity and Thermodilution Derived Indexes of Coronary Physiology
Source: JACC Cardiovasc Interv. 2022 May 23;15(10):1060–70. doi: 10.1016/j.jcin.2022.03.015 (PMC9126183; doi:10.1016/j.jcin.2022.03.015)
Supplement: Supplementary Figures 1 and 2 [file mmc1.docx]

**SUPPLEMENTARY FILE**


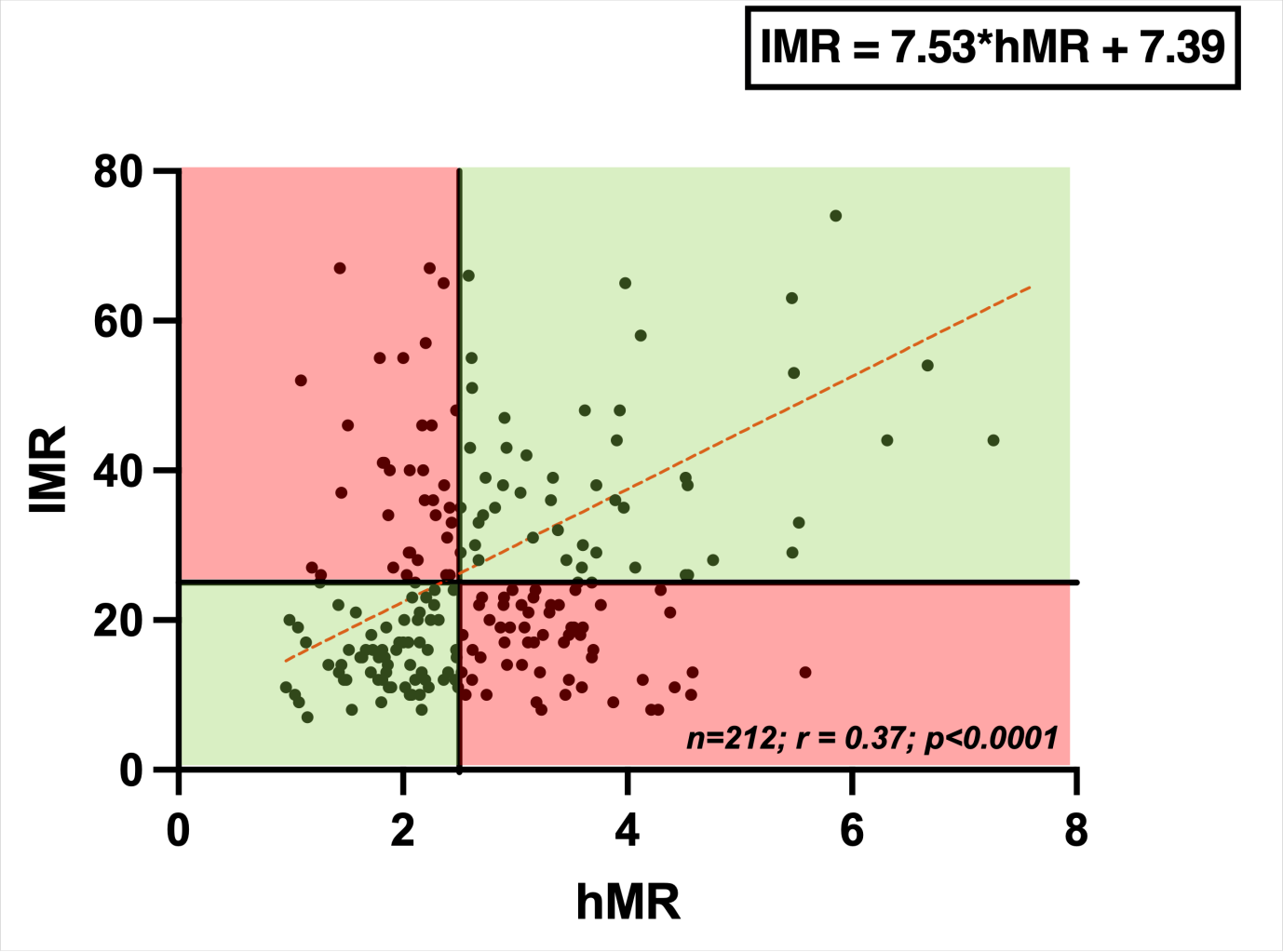


**Supplementary Figure 1.** Scatterplot of hyperemic microvascular resistance (hMR) and index of microvascular resistance (IMR) in patients without significant epicardial coronary artery disease (fractional flow reserve >0.80). The dashed red lines represent the line of best fit - r = 0.37; r^2^ = 0.14; 95% CI 0.24 to 0.48; p <0.001. Quadrants shaded in green have concordant and red discordant measurements, using threshold of hMR ≥2.5 and IMR ≥25.


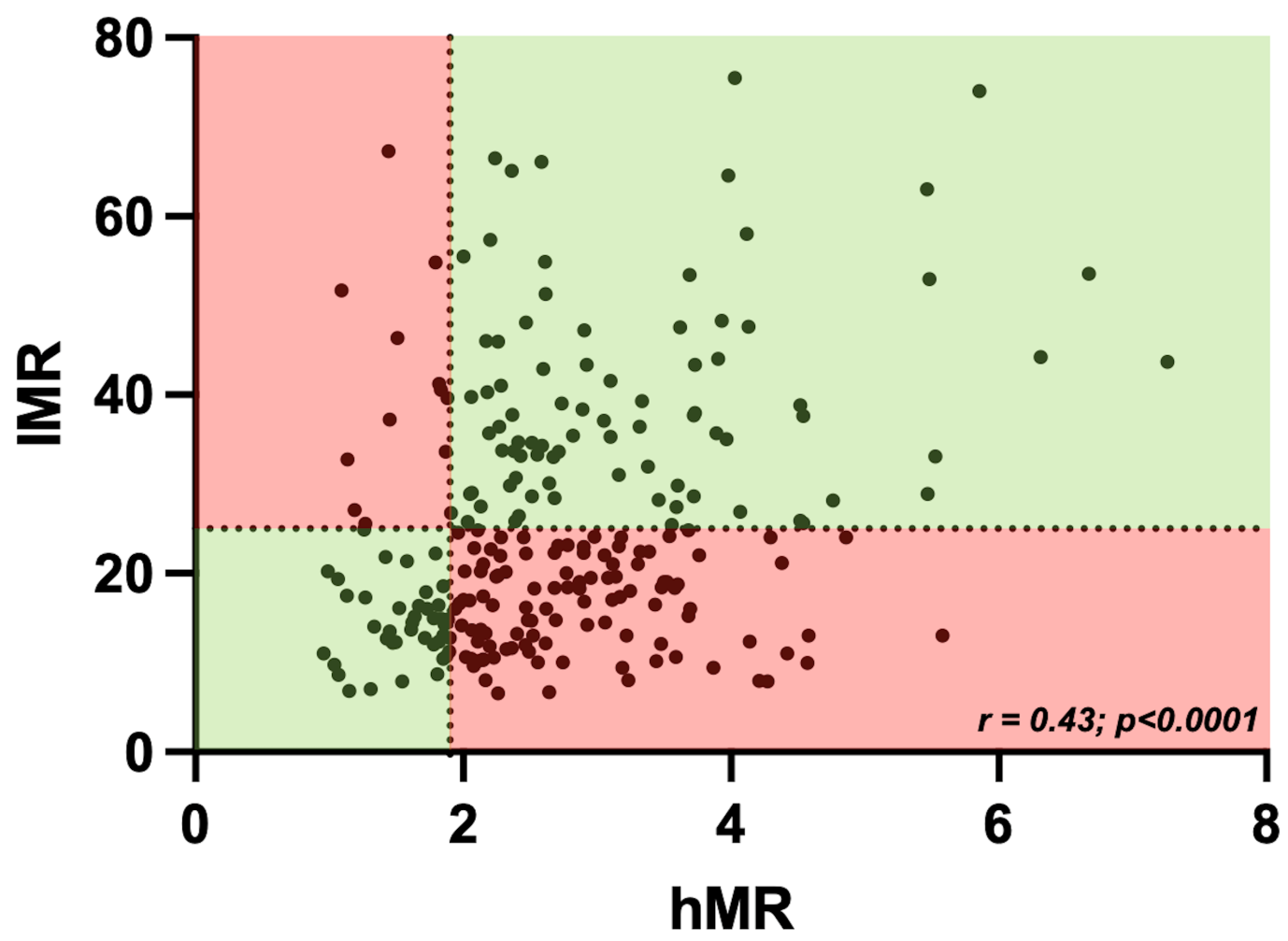


**Supplementary Figure 2.** Scatterplot of hyperemic microvascular resistance (hMR) and index of microvascular resistance (IMR). Quadrants shaded in green have concordant and red discordant measurements, using threshold of hMR >1.9 and IMR ≥25. When dichotomously classified by this new threshold and the commonly used Doppler threshold (hMR >1.9 and IMR ≥25), 189 (78%) hMR and 106 (42%) IMR measurements were abnormal - discordant results were observed in 119 (48%) vessels and concordant results observed in 131 (52%) vessels (concordant 88 abnormal and 43 normal).
